# Biased Trade Narratives and Its Influence on Development Studies: A Multi-level Mixed-Method Approach

**DOI:** 10.1057/s41287-023-00583-z

**Published:** 2023-04-24

**Authors:** Matthias Aistleitner, Stephan Puehringer

**Affiliations:** Institute for Comprehensive Analysis of the Economy (ICAE), Johannes Kepler University of Linz, Altenbergerstraße 69, 4040 Linz, Austria

**Keywords:** Development, Trade narratives, Discourse analysis, Top economic journals, Citation analysis, A14, F13, O19

## Abstract

Recent evidence from citation analysis (Mitra et al., World Dev 135:105076, 2020) suggests that research published in top economic journals is becoming more influential in the development discourse. In this article, we argue that this trend has nontrivial implications for the development discourse on trade in general. Based on an analysis of more than 400 papers published in high-impact economic journals between 1997 and 2017, we highlight three core trade narratives that stand for different biases apparent in the elite economic discourse on trade: “trade championing”, “Ignorance in a world full of nails” and “microfounding trade benefits”. Further insights derived from citation analysis of five development studies journals and a case-study-oriented approach that focusses on the reception of this particular trade debate in World Development suggests that these biased trade narratives are effectively transmitted into development research.

**Supplementary Information:**

The online version contains supplementary material available at 10.1057/s41287-023-00583-z.

## Résumé

Des preuves récentes issues d'analyses de citation [Mitra, S., Palmer, M, Vuon, V. (2020). Développement et interdisciplinarité : une analyse des citations. World Development, 135, 105076] suggèrent que les travaux de recherche publiés dans les meilleures revues économiques deviennent de plus en plus influents dans le récit du développement. Dans cet article, nous soutenons que cette tendance a des implications non négligeables pour le récit du développement relatif au commerce en général. Sur la base d'une analyse de plus de 400 articles publiés dans des revues économiques à fort impact entre 1997 et 2017, nous mettons en évidence trois récits commerciaux fondamentaux qui représentent différents biais apparents dans le discours économique de l'élite sur le commerce : « promouvoir le commerce», « L’ignorance dans un monde plein de clous » et « avantages commerciaux de la micro-fondation ». D'autres informations tirées de l'analyse des citations de cinq revues d'études sur le développement et d'une approche axée sur les études de cas qui se concentre sur la réception de ce débat commercial spécifique dans World Development suggèrent que ces récits commerciaux biaisés sont effectivement transmis dans la recherche sur le développement.

## Introduction

Recent evidence from analyzing three decades of development research challenges the widespread view that development research is an interdisciplinary field of study (Mitra et al. 2020). Taken the subfields of development studies and development economics together, the major share of interdisciplinary communication in terms of citations goes to research published in high-impact generalist economic journals, namely the prominent top 5^1^ (Card and DellaVigna 2013; Heckman and Moktan 2020). Moreover, this share is increasing substantially over time, making these five journals a substantial part of the intellectual foundations of modern development research.

The aim of this article is twofold. First, we aim to provide an in-depth analysis of these particular foundations. More specifically, we use the case of trade research, as an example for a research topic that is of high relevance in both economics and development research. Choosing this case is also motivated by the fact that—at least within (mainstream) economics—there exists a clear consensus position in favor of trade expansion and trade liberalization and its welfare enhancing effects (Irwin 2015; Krugman et al. 2015). This consensus is also evidenced by studies on policy views of economists (e.g. Beyer and Pühringer 2022; Fuller and Geide-Stevenson 2014; Gordon and Dahl 2013)^2^ and more recently, by studies focusing on the economic elite that manifests in research published in top 5 and other prestigious economic outlets (Aistleitner and Pühringer 2021). With regard to the latter, Aistleitner and Pühringer (2021) have also shown that within elite economic trade debate other-than-economic impacts and implications of trade policies (political, social and cultural as well as environmental issues) to a great extent either remain unmentioned or are rationalized by means of pure economic criteria.

Second, we argue that these findings have nontrivial implications for contemporary development research. They suggest that, in addition to its low degree of interdisciplinarity (Mitra et al. 2020), the development discourse draws from a research field that takes an exceptionally narrow perspective when dealing with topics related to the multifaceted challenges of development. By applying a cognitive scientometric approach (Aistleitner et al. 2019; Solla Price 1965) to the case study of trade research we thus aim to clarify whether and to what extent these particular foundations have an impact on development research.

This way, we first aim to show, how research on trade is addressed and framed in professional economic discourses. In doing so, we will highlight the core trade narratives and ‘economic imaginaries’ (Jessop 2013; Sum and Jessop 2013,2013) of trade in current debates among top economists and show how other-than-economic implications and impacts of trade are discursively framed in top economics publications. Second, we then aim to inspect whether and to what extent these trade narratives spill over into the development discourse on trade, using both a more general bibliometric and a case-study-oriented approach.

Methodologically, we follow a multi-level mixed-method approach: In a first step we take a ‘bird’s eye’s view’ and apply a quantitative text analysis of relevant abstracts to inspect the overall evaluation of trade (increases) in the elite economic debate. In a second step, we conduct a qualitative content analysis to highlight the main thematic contexts as well as patterns of argument presented in the discourse revolving around the topic of trade. The qualitative analytical framework, being based on a software-assisted in-depth analysis of discourse fragments, allows us to show how the issue of trade is discursively and rhetorically framed. In a third step, we apply a bibliometric account on the responsiveness of top development study journals to top-level publications in economics and other-than economic disciplines in general and to top-level economic publications on trade in particular. Fourth and finally, we combine the insights of our qualitative analysis and our bibliometric analysis and conduct a case-study of the specific transmission of elite economics trade evaluations and trade narratives into *World Development*, the flagship journal of development studies.

Hence, we will be able to develop a better understanding of prevailing trade narratives in the economics expert debates, may also have an increasing influence on the formation of the development discourse.

The remainder of this article is structured as follows. First, in Sect. "Methodological Framework and Data" we describe our methodological framework including our multi-level mixed-method approach and a description of the data sample. In Sect. "Trade Debates and Narratives in Top Economic Journals" we discuss the results of our empirical analysis of the economic elite discourse comprising a thematic analysis and an analysis of three prevailing trade narratives and imaginaries. Sect. "Influence on Development Studies" provides evidence on the influence of the economic elite discourse on the development discourse based on a citation analysis and a specific case-study. Sect. "Discussion and Conclusion" offers a discussion of our main results and some concluding remarks.

## Methodological Framework and Data

Our analysis of the debate in top economic journals on trade is based on a mixed-method approach combining quantitative methods (*n*-grams and word counts) with a discourse analysis of publications on trade in elite economic and development studies discourse. Whereas the former is applied to inspect the formal structure of the discourse on trade-related issues, a qualitative perspective allows us to identify and examine core imaginaries and narratives in the debate.

Against this background we apply a two-level analysis of discourse comprising a thematic and an in-depth analysis as, for instance, suggested by Krzyżanowski (2010). Whereas in the former step the main discourse topics present in the text are examined, the second step aims to highlight dominant discursive strategies and lines of argumentation. Hence, we employ a discourse analytical approach to unveil core patterns of arguments and discursive strategies in the trade debate in top economic journals and a sample of top development publications. Due to the typically very technical language of academic papers we decided to base our analysis of the trade narrative in economic elite discourse on the abstracts, introductions and conclusions of the papers. We argue that these parts of a paper are a reliable source for the analysis of the trade narrative in economics and development elite discourse because it aims to call attention to the most important information of a paper and should communicate research results to an extended disciplinary community within the profession and beyond (Sala 2014). Yet, since these parts are mainly written in comparably rather plain language, which in turn enables us to apply qualitative analytical methods in the first place.

To obtain representative data of the elite discourse in economics related to trade, we use two different data samples, compiled for a recent paper on the structure of the elite trade discourse in economics (Aistleitner and Pühringer 2021). This sample contains a total of 422 trade-related elite economic papers, among them 372 papers (88%) published in top 5 and 50 highly cited papers published in a set of 16 high-impact economic journals. In all, our sample represents a comprehensive picture of the current discourse about trade in elite economic journals.^3^ Our focus on the elite economic journals is motivated by the observation that economics exhibits strong forms of institutional stratification and a strong internal hierarchy (Bloch et al. 2018; Bühlmann et al. 2017; Schultz and Stansbury 2022). In this context, top economic journals play a decisive role for the structure of the economics discipline. Publications in these journals do not only capture significant parts of the elite discourse (Aistleitner et al. 2019; Fourcade et al. 2015; Gloetzl and Aigner 2019). They also have a strong impact on career trajectories of young economists, define the contemporary methodological and ontological standards of the discipline and thus have a significant influence on future trajectories of the economics discipline (Maesse et al. 2021). That is why many (mainstream) economists are literally obsessed with publishing in one of these journals (Heckman and Moktan 2020; Serrano 2018).

To operationalize the results from our in-depth analysis of trade narratives, we use qualitative codings to analyze the overall normative evaluations of trade and impacts and implications of trade within the trade debate in elite economics (see Tables S1 and S2 in the Supplementary Material). More precisely, we assume that the catchwords outlined in Tables S1 and S2 indicate that a publication shows a distinct overall normative evaluation (i.e. positive, negative, ambivalent or neutral) and addresses distinct impacts and implications of trade and trade expansion (i.e. economic, policy, social and cultural and environmental). While the analysis of impacts and implications of trade is mainly based on the use of distinct catchwords, the normative evaluation of trade is sometimes implicit. Therefore, in order to increase reliability, we both classified the papers separately and developed a common coding system after an initial pre-test, where we discussed uncertain cases. However, still our evaluation was rather cautious and in cases of slightly different classifications of overall trade evaluation, we assigned the respective papers to the category neutral or ambivalent, respectively.

In a consecutive step, we use the codings of evaluation and impacts and implications of trade and applied an in-depth discourse analytical framework to reconstruct typical narratives and economic imaginaries of trade-related issues. While the reconstruction of narratives and imaginaries present in academic publications is not very common particularly in economics, there is strand of literature highlighting the impact of metaphors and language in general also in economics, mainly based on the seminal work of McCloskey’s work on the “rhetoric of economics” (Graupe and Steffestun 2018; Hardt 2014; Klamer 2006; McCloskey 1998). More recently, there is also some research on the role of narratives and disciplinary identities in academic discourse (Hyland 2012, 2018). To sum up, it was argued that the professional authority of the economics discipline and the institutionalization of economics as main policy device, has led to an expansion of a distinct ‘style of economic reasoning’ (problem definitions and the corresponding assumptions, methodological and theoretical approaches and explanations). In this process rather technical economic expert knowledge is being translated into what has been called ‘economic narrative’ or ‘economic imaginary’ (Jessop 2013; Sum and Jessop 2013,2013). These narratives or imaginaries translate complex economic phenomena into a manageable understanding and thus into concrete practices and discourses. Against this background, in this paper we apply a discourse analysis of abstracts (and partly introductions and conclusions) of elite economic publications on trade and thus aim to reconstruct main trade narratives in this discourse. Eventually, we use citation analysis and qualitative content analysis to trace the transmission and reception of these elite economics trade narratives into development research.^4^

## Trade Debates and Narratives in Top Economic Journals

Overall, we found three core patterns of argument in the discourse on trade, which constitute the main elite economists trade narrative(s): The first narrative (‘trade championing’, borrowing a critical remark from Rodrik 2018,5) describes a clear link between the alleged lop-sidedness of economists in favoring trade liberalization in the public debate and the academic debate. The second narrative (‘ignorance in a world full of nails’, referring to the critique against the very narrow economic methodology by Blaug 2001) relates to methodological and conceptual leanings in the profession, which seem to deepen the dominance of a particular trade debate.^6^ The third narrative (‘microfounding trade benefits: the exporting firm’) postulates a positive correlation between a firm’s economic performance and its export orientation. In the following we present our results of main trade narratives in two steps. On the one hand, we make use of our codings on evaluations and impacts and implications of trade and quantitative content analysis of abstracts. On the other hand, we provide illustrative examples from our discourse analysis of the elite economic publications in our sample.

### ‘Trade championing’ in Economic Theory

The first and apparently most dominant discursive pattern in the elite economics trade debate is the overall predominantly supportive stance towards increases in trade volume. Considering the overall evaluation of trade (Table S1) we found that about half of the papers in our sample (48.1%) primarily refer to positive implications of trade while in contrast, about 4.7% report mainly negative implications. Furthermore, 9.0% are coded as ambivalent, as they report positive as well as negative implications of trade. The remaining share of papers (38.2%) takes a rather neutral stance on this issue. Considering the linguistic structure of the debate, typical phrases such as ‘gains from trade’ are among the five most frequent 3-grams in our sample, only exceeded by ‘empirical studies of’, ‘models of trade’, ‘international trade organizations’ and ‘terms of trade’ (see Tables S5 and S6 in the Supplementary File for more detailed results). This high relevance of gains from trade, typically referring to increases in firm or factor productivity or simply efficiency gains in the export sector due to higher competitive pressure, indicates that either the analytical starting point or the overall conclusion of many authors reflects an overall positive stance toward trade expansion. Thus, ‘gains from trade’ serves as a basic concept in economic trade theory, as evidenced by its frequent mention in economics textbooks.^7^

Using a model of sequential production, in which trade induces a reorganization of production that raises domestic productivity, we show that the welfare gains from trade can become arbitrarily large (Melitz and Redding 2014, p. 317).

Aside from this paper entitled ‘Missing Gains from Trade’, there are also many papers in our sample, where authors explicitly addressed and tried to deconstruct the arguments brought forward by critics of globalization and trade volume increases. Sometimes the critique against opponents of further trade liberalization is thus presented on a personal level, where critics are even denied credibility. Referring to high growth rates of China, India, Vietnam and Uganda, e.g. Bhagwati and Srinivasan (2002, p. 182) conclude: “The opponents of trade who allege that it accentuates or bypasses poverty are therefore not credible”.

By choosing a distinct perspective, research question or modeling approach, the authors do not only risk to fall prey to confirmation bias (see also Rodrik 2018, p. 156) but also suggest a distinct interpretation of the (normative) implications of trade. For instance, in Davidson et al. (2012) this is manifested by the view on imperfect institutions such as ‘hampered labor markets’, which prevent globalization-induced improvement of worker–firm-matching and its gains (e.g. productivity increases or reduction of unemployment). Furthermore, as recently also argued by Watson (2017) the still present reference to Ricardo’s comparative advantage concept to demand trade liberalization in poor countries provides an illustrative example of historical and political decontextualization.

To sum up, a very strong discursive pattern in the trade debate in top economic journals is the overall positive normative evaluation of trade and trade expansion. This positive attitude is often not based on empirical observations or modeling but rather used as a common starting point for empirical analysis in economics elite discourse yet at the very beginning of papers in the introduction or the abstract. Against this backdrop, the support for trade liberalization and trade expansion as main source of welfare gains is taken as granted. However, it goes beyond the scope of this paper whether the dominance of a pro-trade narrative is based on an ontological bias of overvaluing overall wealth effects compared to e.g. distributional concerns (which would potentially lead to a publication bias).

### Ignorance in a World Full of Nails

Another main discursive pattern in the economics elite debate on trade is the narrow and often almost exclusive focus on economic causes, implications and impacts of trade. While it is not surprising that economic research is primarily concerned with economic issues, the frequent ignorance towards policy, social or environmental implication of trade expansion, raises concerns, particularly given the over proportional impact of economic research on development studies both on the level of citation flows (Aistleitner 2022; Mitra et al. 2020) as well as on the discursive level (Madrueño and Tezanos 2018). The ignorance of economics towards empirical and theoretical findings of other social sciences though is a long-debated issue the fields of economic sociology (Aistleitner et al. 2019; Fourcade et al. 2015) and philosophy of the social sciences (Dobusch and Kapeller 2009; Mäki 2009). Critical scholars stressed the alleged narrow and ignorant focus of many economists on the one hand as well as the tendency of many scholars beyond economics^8^ to apply theoretical assumptions and economic methodology on other-than economic phenomena and thus coined the label ‘economic imperialism’ (Fine 2002; Mäki 2009).

The narrow perspective in the economic elite discourse about trade in our sample manifests in several ways. First, our coding of trade impacts and implications shows that, unsurprisingly, nearly all papers (95%) referred to the economic impacts and implications of trade. However, slightly more than a fifth (21.8%) of all papers in our sample refer to any kind of social (and cultural) implications of trade. This share seems particularly low, considering that our broadly defined code ‘social and cultural impacts’ comprises various issues from inequality, distribution, migration, employment trajectories, the social welfare state, poverty, social standards or working conditions in trade policy agreements or gender relations (see Table S2). In contrast, most of these issues are addressed in the critical debate on the impacts of trade and globalization in development studies (see below) and other social sciences (e.g. Chang 2009; Crouch 2018; Shaikh 2007). An even more surprising finding of our analysis is that environmental issues are hardly ever addressed in our sample of trade debate in top economic journals, again quite contrary to development studies (e.g. Hickel 2019; Kozul-Wright and Fortunato 2012) and environmental sciences (e.g. Marques et al. 2019; Peters et al. 2011). Overall, only 3.3% of all papers refer to any kind of ecological implications or impacts of trade, thereby confirming recent critique against the neglect of ecological issues in economics in general (e.g. Oswald and Stern 2019; Pestel and Oswald 2021). Political implications and impacts of trade do play a much more important role in our sample. Hence, in a further step we looked closer at the way other-than-economic implications of trade are discursively framed in the economics discourse.

By doing so, we found that papers dealing with political developments or changes in the institutional structure of trade are often solely interpreted against the backdrop of an economic logic and reasoning (e.g. Head 2010). Consequently, in such papers political processes are often reduced to mere market mechanisms in which the actions of interest groups, legislators and the public voters are guided solely by a cost–benefit logic. Another example of a rather idiosyncratic perspective on other-than economic implications of trade policies can be found in the reference to the role of multinational corporations (MNCs) within the global trade regime. MNCs are often criticized by NGOs and anti-globalization movements due to their alleged powerful impact on governments in shaping economic, social and environmental policy to their advantage. In our sample, however, we found 23 abstracts (5.5% of the total sample) including terms^9^ related to MNCs. This relatively small number is already surprising since MNCs are key players in the world economy, shaping the political, social or environmental conditions under which global trade takes place (Vitali et al. 2011). Thus, one would expect a higher share of papers dealing with power issues beyond the standard monopoly/oligopoly model (exceptions include Antràs and Costinot 2010; Holmes et al. 2015). However, only the sociologist Gereffi (1999) indeed provides a more nuanced picture of power issues related to MNCs.

A third and last very telling example in this context is the observation that policy changes, trade agreements or even armed conflicts are interpreted as ‘natural experiments’. The latter is then used as an identification strategy in econometric analysis to test economic (trade) theories. In an influential paper by Pavcnik (2002), for instance, an economic phenomenon (trade liberalization) is completely de-contextualized from its political conditions (the military coup in Chile 1973). Throughout the paper, the author does not refer to any political context of the trade liberalization or their political implications. There is no mention of Pinochet, dictatorship or military at all. Against this background she comes to the conclusion that ‘… in many cases, aggregate productivity improvements stem from the reshuffling of resources and output from less to more efficient producers’ (Pavcnik 2002, p. 245). Apart from providing a telling example for the dis-embeddedness of economic analysis, one may wonder whether taking into account the grave political circumstances in Chile at that time would have changed Pavcnik's analysis and, in particular, her conclusions. Overall, we argue that the economic imperial style of thought leads to a systematic ignorance towards other-than-economic implications but also causes of trade.

### Microfounding Trade Benefits: The Exporting Firm

A third narrative reconstructed from our analysis relates to the ‘exporting firm’ as central research subject. Against the background of an increasing availability of high-quality firm-level and product-level data as well as the more general methodological trend of ‘microfounding’ macroeconomics (see also Kosnik 2015), this particular narrative is supported by our quantitative analysis: First, the words ‘export’ and ‘import’ are among the overall top 10 most frequent used words; Second, issues of trade appear to be discussed less in an ‘international’ and ‘country’-level context while the ‘firm’-level focus gains more prominence over time; And third, ‘quality’ and ‘markup’ are among the top 10 increasing words over time (see Tables S5 and S6 in Supplementary File).

In our qualitative analysis, we also observe a strong positive attribution to exporting firms: exporting is said to be either a result or the cause of properties such as higher productivity, product quality and profitability. This way, it is taken for granted that exporting is closely related to or even a marker for economic success. Against this background a very common introductory statement reads like: ‘the evidence is quite clear [that] good firms become exporters’ (Bernard and Bradford Jensen 1999, p. 1), ‘initially more productive plants [increase] the export share of sales … more than initially less productive plants’ (Verhoogen 2008, p. 489), On the other hand, exporting itself is framed as a beneficial treatment, in particular in a development context.^10^ While the direction of this causality is questioned by earlier papers in our sample (Bernard and Bradford Jensen 1999; Clerides et al. 1998), we observe a tendency of papers finding evidence in favor of the ‘learning-by-exporting’ hypothesis. For instance, Bustos (2011, p. 304) argues that ‘… the increase in revenues produced by trade integration can induce [Argentinian] exporters to upgrade technology’. Moreover, a randomized experiment by Atkin et al. (2017) reveals that ‘learning-by-exporting’ improves the overall productivity and product quality of Egyptian rug producers (including rugs produced for the domestic market). The policy-implications they derive from their study are straightforward:

Given that this learning is induced by demand for high-quality products from knowledge-able buyers in high-income countries, these changes would likely not have occurred as a result of increased market access to domestic markets. (Atkin et al. 2017, p. 611)

Finally, we identify a third and more dynamic component of the narrative of exporting as a marker for success: the resulting efficiency and welfare gains that arise when exporting firms are exposed to competition. In a highly cited paper, Melitz (2003, p. 1695) argues that the competitive pressure at exporting markets induces a selection process to the advantage of the most productive firm. This way, exporting firms facing the constant threat of competition push overall productivity and thus, total welfare.

To sum up, the narrative of the successful exporting firm comprises two mutually reinforcing patterns of argument. On the one hand, many authors, use high-quality firm-level and product-level data to argue that the ‘exporting firm’ is associated with superior economic performance in general. On the other hand, an opening up and liberalization of domestic economies particularly in emerging economies is seen as an opportunity to induce a catching-up process. Hence, trade volume increases, which historically for developing countries typically meant exporting raw materials and primary products, in this narrative is directly related to economic success. Although not always explicitly argued in our sample, this narrative would suggest that opening up of exporting markets is a promising policy implication, particularly for developing countries.

## Influence on Development Studies

After the presentation of the main trade narratives in the economics elite debate, in this section, we aim to explore its reception in development studies (hereafter DS). In doing so, in a first step we discuss more general results from a bird’s eye view on citation patterns in DS. In a second step, we focus on the case of World Development (hereafter WD) as the flagship journal in the field and use the codings as listed in Tables S1 and S2 to apply a network analysis of citation flows to operationalize and map this reception. In a third step, we then highlight the most cited and most citing articles within these networks based on the results of a content analysis of trade evaluations and other-than-economic impacts and implications of trade.

### Impact of (Elite) Economic Trade Research on Trade Research in Development Studies: A Bird’s Eye View

First, we follow the approach of Mitra et al. (2020) and (partially) replicate the analysis of citation flows between five DS journals (World Development, Journal of Development Studies, Development and Change, Development Policy Review and the European Journal of Development Research), the top 5 development economics journals (DevEcon-T5) and the top 5 journals in the core social science disciplines: political science, sociology, geography and anthropology (PSGA-T5). In the context of our specific trade sample, we add three other highly influential economic journals (Journal of Economic Literature, Journal of Economic Perspectives and Journal of International Economics) to the top 5 in economics (Econ-T8) and a selection of 84 ‘environmental’, ‘interdisciplinary’ and ‘multidisciplinary’ journals as an aggregated category (EIM-84; see Tables S3 and S4 in the Supplementary File for a full description of all journals under study). Adding the latter is motivated by the second narrative ‘ignorance in a world full of nails’ (Sect. "Ignorance in a World Full of Nails"), in particular the apparent neglect of environmental impacts and implications of trade.

**Fig. 1:**
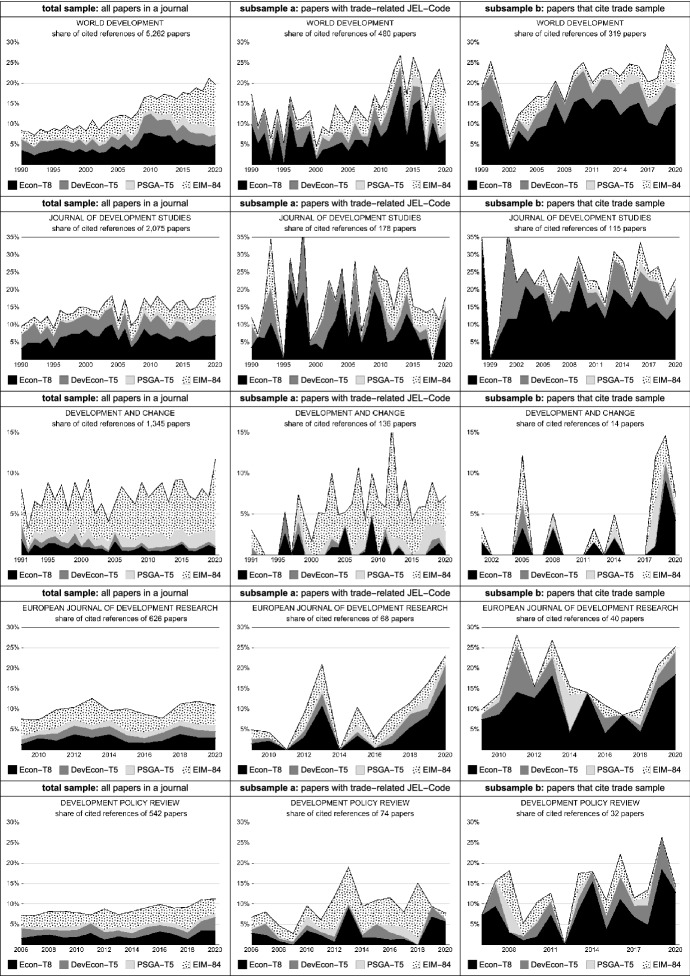
Analysis of citation patterns between five DS journals and selected disciplines/fields. A more detailed description of this analysis is provided in Sect. 2.2 of the Supplementary File

Figure 1 shows detailed results of this analysis and compares cited references in the five DS journals along three different (sub-)samples: All papers published in a journal in the analyzed period (left column), papers that have a trade-related JEL code assigned in EconLit (subsample a) and papers that cite at least one of the papers from our trade sample (subsample b). The observed citation patterns reveal a clear tendency: trade-related papers (subsample a) more frequently cite top economic journals (Econ-T8) than the overall articles in a journal and papers that explicitly cite a paper from our trade sample (subsample b) even more frequently cite Econ-T8 (and also DevEcon-T5). In contrast, references to a broad set of environmental, interdisciplinary or multidisciplinary journals (EIM-84) substantially diminish or at least remain at a relative low level in subsample b. To sum up, these differences in citation patterns across the subsamples suggest differences either in the disciplinary background of the authors and/or in their theoretical orientation—again pointing to an increasing spread of a specific kind of quantitatively oriented economic methodology as discussed in Sect. "Ignorance in a World Full of Nails"—at least in the case of trade-related development issues. In particular, referring to a paper from our trade sample is associated with a narrower reference pattern than papers in WD that deal with trade issues but do not cite a paper form our sample at all. Finally, a notable exception from this overall trend is Development and Change which not only hardly cites papers from our trade sample (between 2001 and 2020 only 14 papers in absolute terms) but also refers significantly less to top economic journals in subsample b [in contrast, we can observe a pronounced interdisciplinary citation pattern including a stronger reference to other social science disciplines (PSGA-T5)].

### Mapping the Reception of Economic Narratives via Citation Networks: The Case of World Development

**Fig. 2:**
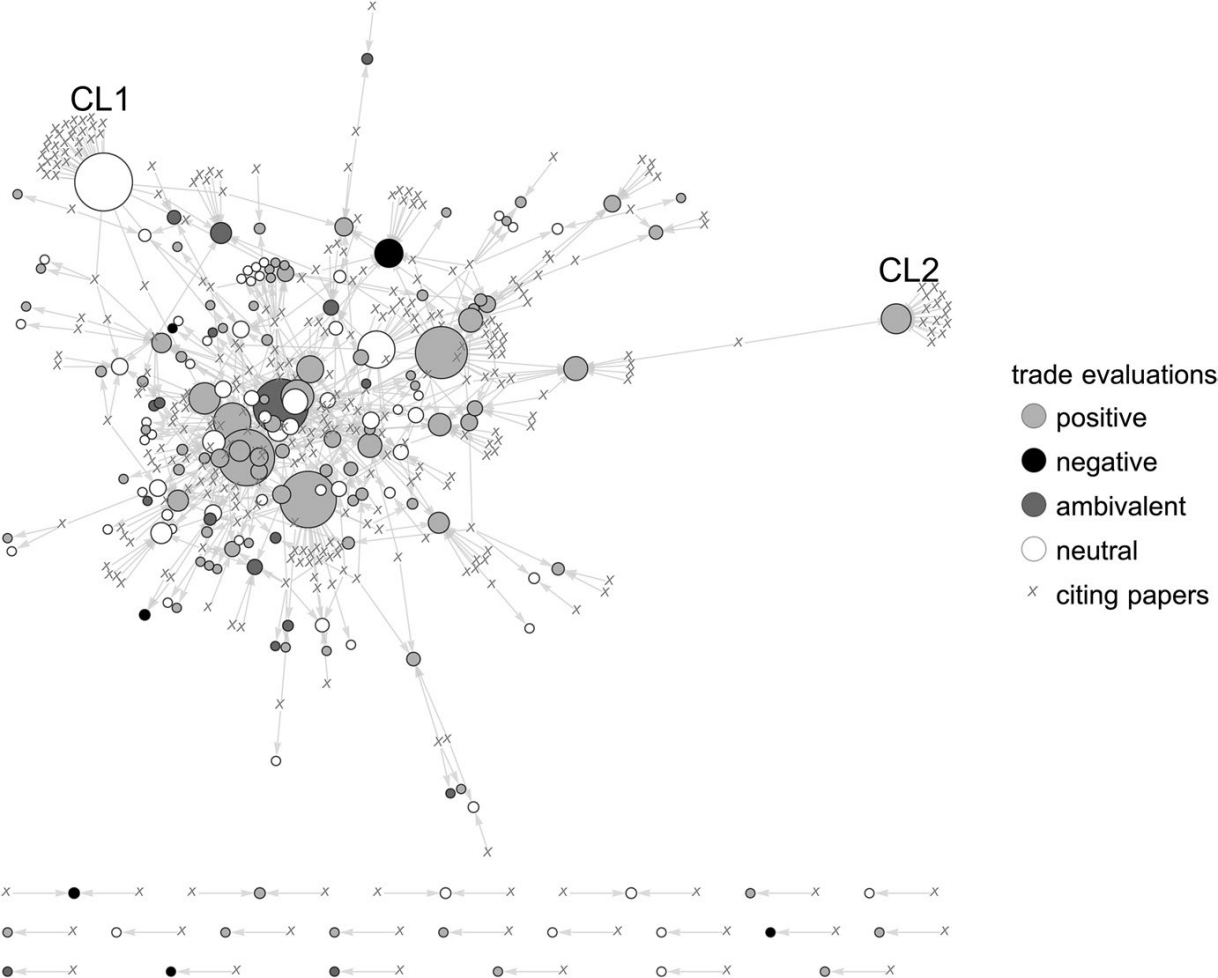
Citation network between World Development and the elite economics trade debate: cited trade evaluations

**Fig. 3:**
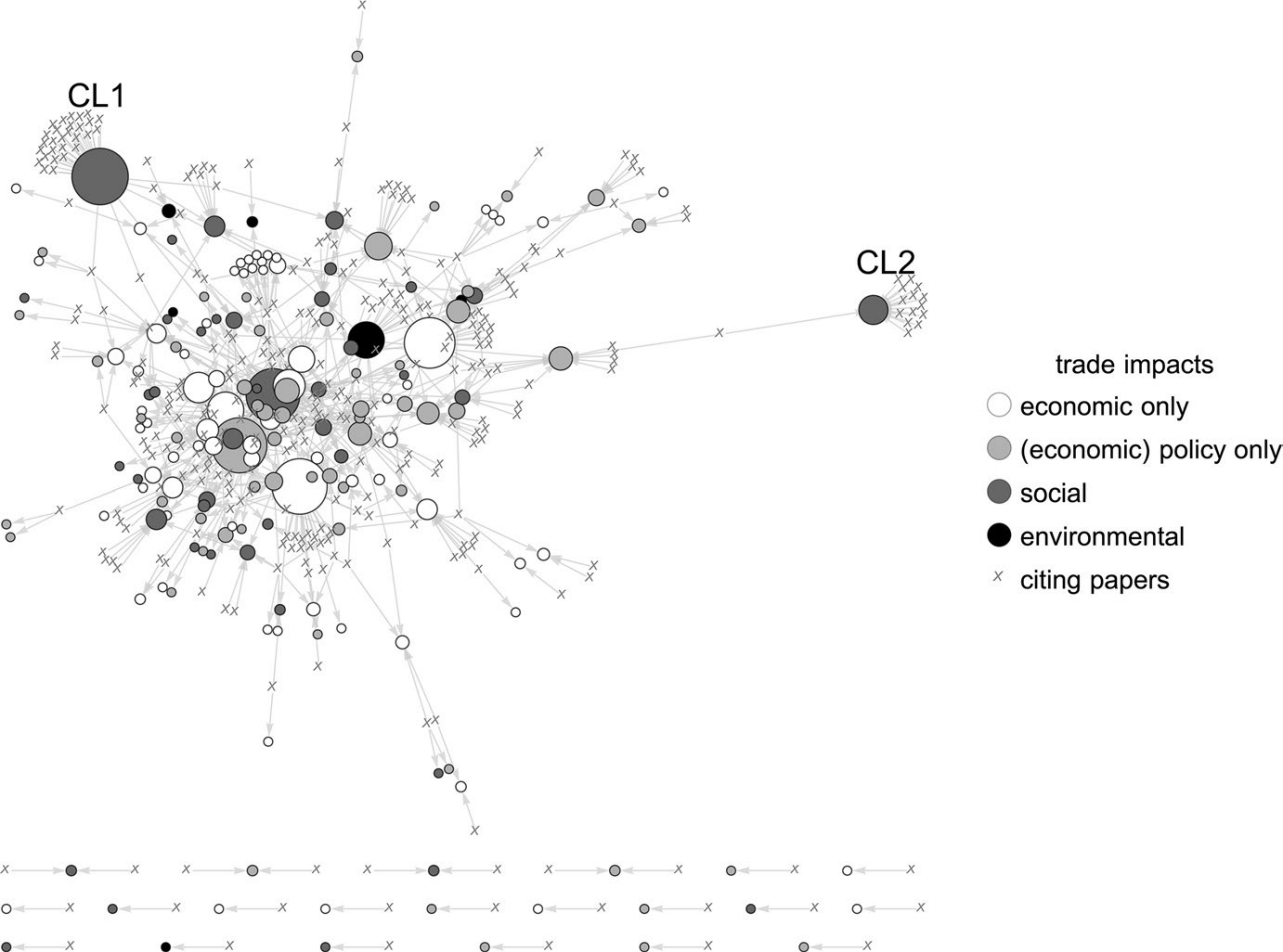
Citation network between World Development and the elite economics trade debate: cited trade impacts

Our further analysis is restricted to WD, the flagship journal in DS (Madrueño and Tezanos 2018; Mitra et al. 2020), where we analyze the citation linkages between articles published in the journal and the articles in our trade sample.^11^ More specifically, we plot a directed citation network (citing article → cited article) with the vertex size being weighted by its in-degree centrality (i.e. the number of its citations). Here, we map the network by shading the cited articles according to its overall trade evaluation (Fig. 2) and its discussion of trade impacts and implications (Fig. 3).

While the overall structures of the network indicates that many of the most cited papers are cited *together*, on average, half of all papers in our trade sample are not cited at all. In total, 323 WD papers cite 179 papers (42.4%). This means that this specific part of the development discourse is strongly influenced by a relatively small set of economic elite papers. Moreover, two notable exceptions (see cluster CL1 and CL2 in Figs. 2, 3) are both papers that take a special position within the elite economic trade debate. CL2 forms around Collier and Gunning’s (1999) review on the explanations of poor economic performance of African countries (including, among others, the lack of openness to international trade). CL1 mirrors a larger discourse that centers around a specific agenda in development research: the global value chain (GVC) approach, represented by Gereffis’ (1999) seminal analysis of the apparel industry and its embeddedness in international trade networks.

The most obvious explanation for this citation flows may be that a substantial share of research published in elite economics on the issue of trade (policies) is simply not relevant for the development discourse. However, a more nuanced explanation could be that—due to the observed pro-trade bias within the debate—a major share of papers is not being taken up in the development discourse on trade, while in turn more critical or ambivalent contributions receive comparatively more attention and are thus cited more frequently. Yet, Fig. 2 shows, that a major share of citations goes to papers that either take a positive or neutral stance toward the effects of trade. More specifically, 57.5% of all citations go to papers with a positive trade evaluation while only 12.6% of all citations go to papers with either a negative or ambivalent evaluation.

Put differently, although a substantial share of papers is neglected in the development discourse on trade, in terms of citations, the pro-trade bias immanent in elite economics receives substantial attention in WD.

In a similar vein, this analysis also supports the evidence derived from the analysis of overall citation patterns (Sect. "Impact of (Elite) Economic Trade Research on Trade Research in Development Studies: A Bird’s Eye View") and shows a considerable reception of a far-reaching ignorance towards other-than-economic implications and impacts of trade from elite economic discourse into development research (Fig. 3). Again, the very low reference to environmental impacts and implications of trade (which are already poorly represented within the sample) reveals that economic elite publications on trade hardly contribute to critical debates regarding the (detrimental) effects of trade on the environment. In all, the majority of economic papers that have an impact on development focus either on pure economic or (economic) policy impacts and implications of trade.

#### The Top Cited Papers

Table 1 lists the 43^12^ most cited papers in WD with their respective codings. Only one paper with a negative and two with an ambivalent evaluation are among this set of papers. Almost two thirds of the papers take a positive stance towards trade. In terms of trade impacts, again more than two thirds of the papers discuss merely economic (17 papers) or economic and policy implications of trade (13 papers).

**Table 1:** The 43 most cited papers of the economic trade debate in WD

Rank	Source	Source title	Journal	Trade evaluation	Trade impacts
1	Gereffi (1999)	International trade and industrial upgrading in the apparel commodity chain	J Int Econ	Neutral	Econ, Soc
2	Melitz (2003)^‡^	The impact of trade on intra-industry reallocations and aggregate industry productivity	Econometrica	Positive	Econ, Pol
3	Javorcik (2004)	Does foreign direct investment increase the productivity of domestic firms? In search of spillovers through backward linkages	Am Econ Rev	Positive	Econ
4	Fischer (2003)*	Globalization and its challenges	Am Econ Rev	Ambivalent	Pol, Soc
5	Frankel and Romer (1999)	Does trade cause growth?	Am Econ Rev	Positive	Econ
6	Bernard and Jensen (1999)^‡^	Exceptional exporter performance: cause, effect, or both?	J Int Econ	Positive	Econ
7	Copeland and Taylor (2004)	Trade, growth, and the environment	J Econ Lit	Neutral	Pol, Env
8	Anderson and Van Wincoop (2003)	Gravity with gravitas: A solution to the border puzzle	Am Econ Rev	Positive	Econ
9	Clerides et al. (1998)^‡^	Is learning by exporting important? Micro-dynamic evidence from Colombia, Mexico, and Morocco	Q J Econ	Positive	Econ
10	Collier and Gunning (1999)	Explaining African economic performance	J Econ Lit	Positive	Econ, Pol, Soc
11	Rodrik (1998)	Why do more open economies have bigger governments?	J Polit Econ	Negative	Econ, Pol
12	Anderson and Van Wincoop (2004)	Trade costs	J Econ Lit	Positive	Econ
13	Helpman et al. (2008)	Estimating trade flows: Trading partners and trading volumes	Q J Econ	Neutral	Econ, Pol
14	Limao and Venables (2001)	Infrastructure, geographical disadvantage, transport costs, and trade	World Bank Econ Rev	Positive	Econ, Pol
15	Wacziarg and Welch (2008)	Trade liberalization and growth: New evidence	World Bank Econ Rev	Positive	Econ, Pol
16	Edwards (1998)	Openness, productivity and growth: What do we really know?	Econ J	Positive	Econ, Pol
17	Imbs and Wacziarg (2003)	Stages of diversification	Am Econ Rev	Neutral	Econ
18	Dollar and Kraay (2003)	Institutions, trade, and growth	J Monet Econ	Positive	Econ, Pol
19	Verhoogen (2008)^‡^	Trade, quality upgrading, and wage inequality in the Mexican manufacturing sector	Q J Econ	Neutral	Econ, Pol, Soc
20	Van Biesebroeck (2005)^‡^	Exporting raises productivity in sub-Saharan African manufacturing firms	J Int Econ	Positive	Econ
21	Bernard et al. (2003)^‡^	Plants and Productivity in international trade	Am Econ Rev	Positive	Econ, Pol, Soc
22	Hummels and Klenow (2005)	The variety and quality of a nation’s exports	Am Econ Rev	Neutral	Econ
23	Wood (1997)	Openness and wage inequality in developing countries: The Latin American challenge to East Asian conventional wisdom	World Bank Econ Rev	Ambivalent	Econ, Pol, Soc
24	Griffith et al. (2004)	Mapping the two faces of R&D: Productivity growth in a panel of OECD industries	Rev Econ Stat	Positive	Econ
25	Head et al. (2010)^†^	The erosion of colonial trade linkages after independence	J Int Econ	Positive	Econ
26^#^	Bernard et al. (2007)^‡^	Firms in international trade	J Econ Perspect	Positive	Econ
27^#^	Amiti and Konings (2007)	Trade liberalization, intermediate inputs, and productivity: Evidence from Indonesia	Am Econ Rev	Positive	Econ, Pol
28^#^	Pavcnik (2002)^†^	Trade liberalization, exit, and productivity improvements: Evidence from Chilean plants	Rev Econ Stud	Positive	Econ
29^#^	Krueger (1997)*	Trade Policy and economic development: How we learn	Am Econ Rev	Positive	Econ, Pol, Soc
30^#^	Eaton and Kortum (2002)	Technology, geography, and trade	Econometrica	Positive	Econ, Pol
31^#^	Helpman et al. (2004)^‡^	Export versus FDI with heterogeneous firms	Am Econ Rev	Neutral	Econ
32^#^	Schott (2004)	Across-product versus within-product specialization in international trade	Q J Econ	Neutral	Econ, Pol, Soc
33^#^	Baier and Bergstrand (2007)	Do free trade agreements actually increase members' international trade?	J Int Econ	Neutral	Econ, Pol
34^#^	Rauch and Trindade (2002)	Ethnic Chinese networks in international trade	Rev Econ Stat	Neutral	Econ, Soc
35^#^	Alcala and Ciccone (2004)	Trade and productivity	Q J Econ	Positive	Econ, Pol
36^#^	Rauch (1999)	Networks versus markets in international trade	J Int Econ	Neutral	Econ
37^#^	Nunn (2007)	Relationship-specificity, incomplete contracts, and the pattern of trade	Q J Econ	Neutral	Econ, Pol
38^#^	Anderson and Marcouiller (2002)	Insecurity and the pattern of trade: An empirical investigation	Rev Econ Stat	Positive	Pol, Soc
39^#^	Chinn and Ito (2006)	What matters for financial development? Capital controls, institutions, and interactions	J Dev Econ	Positive	Econ, Pol
40^#^	Hummels et al. (2001)	The nature and growth of vertical specialization in world trade	J Int Econ	Neutral	Econ
41^#^	Yeaple (2005)	A simple model of firm heterogeneity, international trade, and wages	J Int Econ	Positive	Econ
42^#^	Rauch (2001)	Business and social networks in international trade	J Econ Lit	Positive	Econ
43^#^	Bhagwati and Srinivasan (2002)*	Trade and poverty in the poor countries	Am Econ Rev	Positive	Econ, Soc

The ranking in the first column is based on the sum of the in-degree centralities of the cited papers in the WD citation network. Papers that are designated in the Source column as follows stand for archetypical examples of the trade narratives discussed in Sect. "Influence on Development Studies": *‘trade championing’, ^†^‘ignorance in a world full of nails’, and ^‡^‘microfounding trade benefits: the exporting firm’

Most strikingly, however, is the high prevalence of papers in Table 1 that we use in Sect. "Trade Debates and Narratives in Top Economic Journals" to illustrate the specific trade narratives we identified during our analysis. This holds in particular for ‘microfounding trade benefits: the exporting firm’, confirming its high influence within the development discourse on the issue of trade and globalization.

#### The Top Citing Papers

While these results so far suggest a significant reception of the trade narratives from elite economic discourse into the development discourse, they do not tell us anything about the context in which these narratives are discussed. Citing a paper does not necessarily translate into agreeing with it, but may also express signaling awareness of the literature, critical engagement or even disagreement. To confront this argument, we apply our coding approach, described in Sect. "Methodological Framework and Data" to the most citing papers within our citation network (see Table S7). More specifically, we measure the *Out-degree centrality* (denoting articles that are citing multiple times) of the vertices in Figs. 2 and 3 and select the top 30 citing articles in WD.^13^

**Fig. 4:**
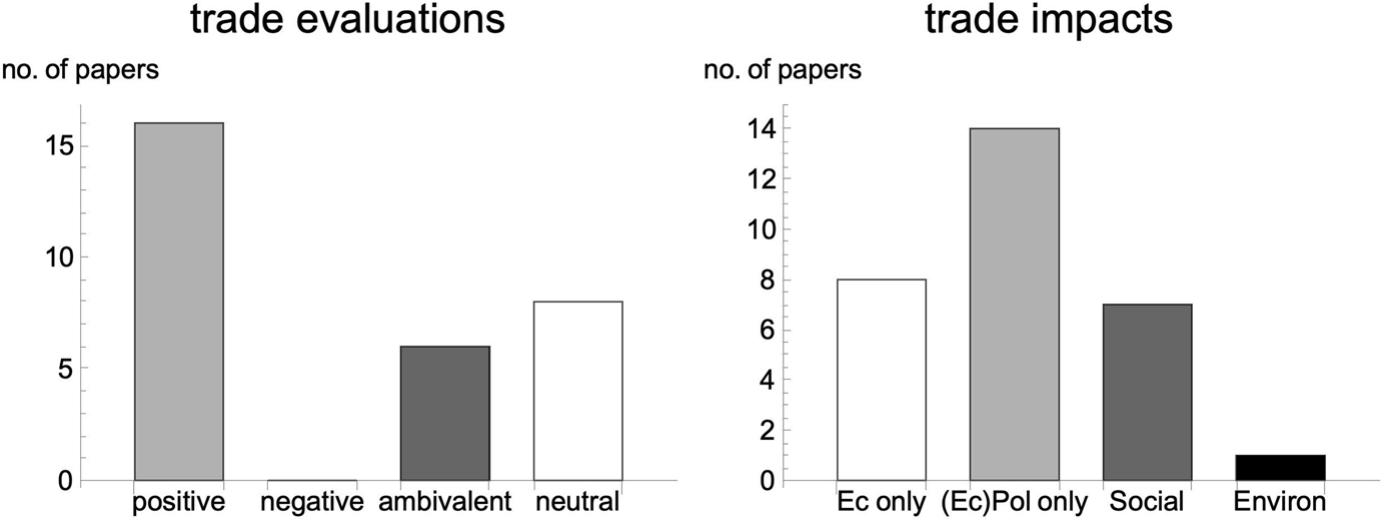
Normative trade evaluations and trade impacts/implications of the top 30 citing papers in WD

Figure 4 gives an overview of the results of this analysis by plotting the respective codings: trade evaluations and trade impacts.

At least, within this subsample of papers, a quite similar pattern emerges. Overall, a clear pro-trade bias becomes visible while in turn there is no paper within this set of citing papers that addresses solely negative effects of trade expansion. However, looking at the top ranked citing papers in Table S7, still reveals a somewhat nuanced picture: Here, a substantial share of papers that most frequently cite our sample of elite economic trade discourse take a neutral stance on trade. Moreover, three out of six ambivalent papers are ranked among the top 10. Thus, although our results indicate a considerable influence of a pro-trade bias from the elite economics discourse, there are many examples of a critical engagement with the findings of economic research (e.g. Francois and Manchin 2013; Goel 2017). In terms of policy impacts and implications of trade, results are also more balanced. However, in line with the general pattern observed within the elite economics trade debate, environmental impacts still play a neglected role in these papers.

To sum up, the results of our analysis of the most citing papers in WD reveal a strong resemblance with the elite economics trades debate. This is in line with Mitra et al. (2020) who find that, due to increasing competitive pressures, economists themselves tend to publish more in development journals. We also confirmed this by an analysis of the CV of the authors of the top 30 citing articles in WD, where we found that the majority of them obtained their PhD in economics (50 out of the 66 authors listed in Web of Science). Consistent with previous research on the marginalization of Southern development researchers (Amarante and Zurbrigg 2022; Cummings and Hoebink 2017) we also found that almost half of all authors (45.5%) graduated at an Anglo-Saxon institution while the remaining institutions we were able to identify (40.9%) are located in European countries.

## Discussion and Conclusion

Our article aims to examine the influence of trade narratives in elite economic journals on DS. Hence, we apply a multilevel mixed-method approach and combine quantitative text analysis with a discourse analytical approach to examine dominant narratives and imaginaries present in high-impact papers dealing with trade and trade-policy issues. By drawing on a multi-dimensional set of codings that enable an operationalization of this discourse we then apply a citation analysis as well as an in-depth analysis of selected most-cited development papers to demonstrate how these narratives are channeled into the development debate. For this purpose, we use WD as representative outlet where we subsequently apply our discourse analytical framework to a sample of the most citing papers. Hence, with this article we contribute to the literature on the development discourse by providing, first, an in-depth analysis of an essential and constantly growing part of its intellectual foundations and, second, an in-depth mixed-method analysis of those papers within the development discourse that most frequently relate to these foundations.

It should be emphasized that our scientometric approach focusses specifically on the development discourse which refers to the elite economic debate on trade. However, given the recent evidence in terms of citation patterns (Mitra et al. 2020), we argue that we do not only have uncovered a potential source of controversy that surrounds the globalization debate in general, but also indications of a discursive trend in development research that may be problematic for at least two reasons.

First, compared to other social scientists, economists tend to interpret citations more strongly as an adequate proxy for scientific quality (Aistleitner et al. 2019; D'Ippoliti 2021; Fourcade et al. 2015). However, as D'Ippoliti (2021) demonstrates, citations might also serve as an indicator for social and ideological proximity (see also Colussi 2018; Goyal et al. 2006), a circumstance which inevitably provokes critical reflections regarding the role of ideology and political preferences in economic research (see also Beyer and Pühringer 2022; Clark 2017; Javdani and Chang 2023).

Second, the case of trade (policy) research suggests that, by drawing (increasingly) on research published in these high-impact economic outlets, the development discourse also ‘imports’ properties of the economic mainstream that have been subject to a longstanding and critical debate within the social sciences (e.g. Chang 2009; Dobusch and Kapeller 2009; Rodrik 2018; Gräbner et al. 2020). While ‘trade championing’ and ‘ignorance in a world full of nails’ point to more fundamental biases in ideological and epistemological terms (in particular we refer to the theory-ladenness of observation and measurement Kuhn 1970), ‘microfounding trade benefits: the exporting firm’ implies a straightforward policy agenda of trade liberalization whose efficacy and impacts for development are subject to various controversial debates (e.g. Crossa and Ebner 2020; Hou et al. 2022; Langer and Stewart 2012; Lebdioui 2019). It is important to note that a more balanced and maybe also critical discourse towards trade could take place in other economic journals and subfields of economic research. However, again we want to emphasize the enormous importance of top journals for the stratification logics in economics.

Notwithstanding these results, it should be further stressed that most (if not all) of the academic debate’s influence on actual trade policy is, at best, implicit. Real-world policy making is a complex process and decisions are mainly influenced by many different factors and actors (e.g. in a certain country), aside from research and policy ideas (Chwieroth 2010; Cormier and Manger 2022). However, an additional analysis of the institutional affiliations of authors that publish trade-related research in our sample of DS journals (see Fig. S1 in the Supplementary File) indicates at least one additional (aside from being an influential “top 5” economic author) and potential channel of policy influence as the World Bank and the IMF—assumingly two of the most important international policy institutions in the field of trade—appear as the most frequent affiliations. Hence, the question of policy impact of trade research remains a fruitful avenue for future research.

To sum up, with the analysis of the economics elite debate on trade (policies) we have uncovered the case of a highly influential research field that takes an exceptionally narrow perspective when dealing with topics related to the multifaceted challenges of development. This, in turn, suggests that rather than focusing on whether or not DS is an interdisciplinary field of study, it should critically reflect its disciplinary trajectory that comes with an increasing engagement with elite mainstream economics.

### Supplementary Information

Below is the link to the electronic supplementary material.

Supplementary file1 (DOCX 190 KB)

## Data Availability

All data and samples used for the quantitative analysis of the elite trade debate and the analysis of citation patterns can be found in the supplementary file. All further data are available upon request.

## Footnotes

1 The American Economic Review, the Quarterly Journal of Economics, the Journal of Political Economy, Econometrica and the Review of Economic Studies.

2 This way, only about 5% of the respondents of a survey among economists disagreed with the statement that ‘tariffs and import quotas usually reduce general welfare’ (Fuller and Geide-Stevenson 2014, p. 134).

3 For a complete description of the sample see Aistleitner and Pühringer (2021).

4 Further details on the methodological strategy of our study can be obtained from Sect. 2 in the article’s Supplementary Material.

5 ‘It has long been an unspoken rule of public engagement for economists that they should champion trade and not dwell too much on the fine print’ (Rodrik 2018, p. x).

6 Blaug (2001, p. 152) ironically stated: ‘Of course, if a person has a hammer, everything looks like a nail and if an economist has modern tools, then every issue looks like a chance to apply those tools’.

7 We are grateful to an anonymous reviewer for pointing to this aspect. Indeed, searching for the term ‘gains from trade’ in Krugman and Obstfeld’s (2002) standard textbook on ‘International Economics’ reveals 48 results, in the 12th global edition (Krugman et al. 2023) even 62 results (excluding table of contents and index). Another example is volume 4 of the ‘Handbook of International Economics’ with 124 results (Gopinath et al. 2014). It should be noted, that most of the editors and authors of these books are also authors in our sample.

8 This ignorance towards findings from other disciplines often manifests in a rather subtle way as economists may still refer to research from outside their discipline (e.g. political sciences).

9 Our search terms included ‘multinational’, ‘transnational’, ‘production chain’, ‘commodity chain’, and ‘production network’.

10 A notable exception in our sample is Mayer et al.(2014) who examine the performance effects of French exporting firms.

11 Citation data was retrieved on 4 March 2021 from Web of Science.

12 In fact, we list 43 papers since ranks 26–43 are ranked equally in terms of citation frequency.

13 In doing so, it should be stressed that we focus only on papers that most intensively refer to the elite economics debate on trade. Since there are many more papers in WD that cite the papers in our sample, this exercise allows only cautious conclusions regarding the potential reception of elite economic narratives.
