# Supplementary material for: Biased Trade Narratives and Its Influence on Development Studies: A Multi-level Mixed-Method Approach
Source: Eur J Dev Res. 2023 Apr 24;35(6):1322–46. doi: 10.1057/s41287-023-00583-z (PMC11116101; doi:10.1057/s41287-023-00583-z)
Supplement: Supplementary file 1 — Supplementary file1 (DOCX 190 KB) [file 41287_2023_583_MOESM1_ESM.docx]

Biased trade narratives and its influence on development studies. A multi-level mixed-method approach.

Matthias Aistleitner^a*^ and Stephan Puehringer^a^

^a^ Institute for Comprehensive Analysis of the Economy (ICAE)
 Johannes Kepler University of Linz, Austria

*Corresponding author. Email: matthias.aistleitner@jku.at

Supplementary information

# Introduction

This file provides complementary information to the article “Biased trade narratives and its impact on development studies”. Its purpose is to provide information on specific methodological aspects (section 2) as well as to present further and more detailed outputs of the data used in our study (sections 3–5).

# Methodological framework

## Description of qualitative codings

To operationalize the results from our in-depth analysis of trade narratives, we use qualitative codings to analyse the overall normative evaluations of trade (Table S1) and impacts and implications of trade (Table S2) within the trade debate in elite economics and a sample of the 30 elite development studies publications, most referring to the economic elite debate in terms of citations (see also section 4.2). Although we basically used the abstracts for the coding of the papers, we included the full papers in cases where we could not decide about a coding on the basis of an abstract and/or when the abstracts were very short.

*Table S1. Trade evaluations*

| **Code** | **Related topics** |
| --- | --- |
| *positive* | e.g. references to efficiency gains, welfare, productivity or product quality increases, the theory of comparative advantage |
| *negative* | e.g. increases in unemployment, poverty, negative distributional or environmental effects of trade increase |
| *neutral* | without any kind of normative evaluation |
| *ambivalent* | mix between positive and negative |

*Table S2. Implications and impacts of trade*

| **Code** | **Related topics** |
| --- | --- |
| *economic* | e.g. prices, cost structures, productivity, market structures, export/import quotas, firm productivity |
| *policy* | e.g. tariffs, custom unions, trade agreements, policy institutions, liberalisation and protectionism, trade barriers, government interventions |
| *social and cultural* | e.g. changes in employment/income, living- and working-conditions of workers; class, gender, ethnicity and/or cultural background of workers |
| *environmental* | e.g. ecological impacts of trade (emissions, environmental pollution, ecosystem), environmental trade policies, carbon taxing |

## Analysis of citation patterns

To trace the transmission and reception of elite economics trade narratives into development research, we provide an extensive analysis of citation patterns and classify cited references in five development studies (DS) journals by combining two different strategies. First, we follow Mitra et al. (2020) and (partially) replicate the analysis of citation flows between five DS journals, five development economics journals and the top 5 journals in five core social science disciplines (see Table S3 for a detailed journal list). For the selection of the five DS journals, we deviate from Mitra et al. (2020) as the Journal of Human Development and Capabilities (JHDC) has published only few papers related to trade (as defined in our article; see below for further details). Instead of the JHDC, we selected EJDR as the fifth journal. In the context of our specific trade sample, we added three other highly influential economic journals (JEL, JEP and JIE) to the top 5 economic journals. In Figure 1 in the main article, we aggregate these eight journals in economics (Econ-T8) as well as the four other core social science disciplines (PSGA-T5).

*Table S3. Selection of journals and their disciplinary classification based on the approach of Mitra et al. (2020).*

| **Discipline/field** | **Journal** |
| --- | --- |
| Development Studies (DS) | World Development (WD)  Journal of Development Studies (JDS)  Development and Change (DECH)  Development Policy Review (DPR)  European Journal of Development Research (EJDR) |
| Development Economics (DevEcon-T5) | Journal of Development Economics  World Bank Research Observer  World Bank Economic Review  Economic Development and Cultural Change  Review of Development Economics |
| Economics  (Econ-T8) | Quarterly Journal of Economics  Econometrica  Journal of Political Economy  Review of Economic Studies  American Economic Review |
|  | Journal of Economic Literature  Journal of Economic Perspectives  Journal of International Economics |
| Political Science  (PSGA-T5) | International Organization American Journal of Political Science  Annual Review of Political Science  American Political Science Review  British Journal of Political Science |
| Sociology (PSGA-T5) | American Sociological Review  American Journal of Sociology  British Journal of Sociology  Social Problems  European Sociological Review |
| Geography (PSGA-T5) | Global Environmental Change – Human and Policy Dimensions  Economic Geography Progress in Human Geography Journal of Economic Geography  Cambridge Journal of Regions Economy and Society |
| Anthropology (PSGA-T5) | American Ethnologist Current Anthropology American Anthropologist  Annual Review of Anthropology  Cultural Anthropology |

Second, we use an additional classification of Aistleitner (2022) who provides information of the disciplinary background of 84 influential journals related to the categories ‘environmental’, ‘interdisciplinary’ and ‘multidisciplinary’ journals (see Table S4; in Figure 1 we also aggregate these journals into one category (EIM-84)). For the analysis of citation patterns, we match bibliometric data from the Web of Science (WoS) database and the EconLit database. While WoS contains information on cited references for each article, Econlit provides more detailed information on author affiliations (see below) and JEL-Codes (EconLit search category “subjects”) for each article. By doing so, we are able to compare the cited references in the five DS journals along three different (sub- )samples (as shown in Figure 1): All papers published in a journal in the analysed period (left column), articles that have a trade-related JEL code assigned in EconLit (“subsample a” column) and articles that cite at least one of the papers from our trade sample (“subsample b” column). For the definition of “subsample a” we selected every article that contains one of the following strings in the EconLit “subjects” field: “trade”, “international economic order and integration”, “economic integration”. “open economy macroeconomics” and “economic growth of open economies”.

*Table S4. Selection of journals and their disciplinary classification based on Aistleitner (2022).*

| **Discipline/field** | **Journal** | |
| --- | --- | --- |
| Environmental (EIM-84) | Land Use Policy  Ecology and Society  Conservation Biology  Environment and Urbanization  Climatic Change  Environmental Science and Policy  Environmental Conservation Agriculture Ecosystems and Environment  Ambio  Resources Policy  Environmental Research Letters  Environmental Management  Nature Climate Change  International Forestry Review  Journal of Environmental Management  Journal of Cleaner Production  Annual Review of Environment and Resources  Conservation and Society  Biological Conservation  Forest Ecology and Management | Climate Policy  Current Opinion in Environmental Sustainability  Agroforestry Systems  Water Policy  Conservation Letters  International Journal of the Commons  Mountain Research and Development  Environment: Science and Policy for Sustainable Development  Wiley Interdisciplinary Reviews-Climate Change  Natural Resources Forum  Regional Environmental Change  Oryx  Sustainability  Water Resources Research  Forests  Water Alternatives  Water International |
| Interdisciplinary (EIM-84) | Ecological Economics  Economic and Political Weekly  Social Science and Medicine  Energy Policy  Journal of Modern African Studies  Feminist Economics  China Quarterly  Africa  Journal of Agrarian Change  Journal of Developing Areas  Bulletin of Indonesian Economic Studies  European Journal of Political Economy  Disasters  Journal of Economic History  Economics and Politics  Marine Policy  Studies in Family Planning | Review of African Political Economy  Asian Survey  Agriculture and Human Values  Journal of Southern African Studies  Industrial and Labor Relations Review  Habitat International  Technological Forecasting and Social Change  Climate and Development  Policy Sciences  Social Science Quarterly  Population and Environment  Latin American Politics and Society  Journal of Policy Analysis and Management  Signs  Journal of Regional Science |
| Multidisciplinary (EIM-84) | Food Policy  Science  PNAS  Nature  Forest Policy and Economics  PLOS One  International Labour Review  Agricultural Systems  Latin American Research Review | Urban Studies  Journal of Latin American Studies  Annals of the American Academy of Political and Social Science  African Studies Review  Comparative Studies in Society and History  Journal of Asian Studies |

# Trade debates and narratives in top economic journals

To get a first thematic overview of the debate, we looked at lemmatized word frequencies and n-grams based on the article’s abstracts (see Table S5 and S6). It should be noted, however, that a substantial share of the papers which enter our final analysis do not contain an abstract. Where available, we used abstract of the papers listed in databases such as AEAweb, RePec, ResearchGate etc. This way, we ended up with 395 abstracts for our sample of 422 papers. For the remaining cases, we compiled “pseudo-abstracts” and analysed those first paragraphs (and if necessary, the conclusion) of a paper until we were able answer three main questions which define an abstract: i) why the research was conducted, (ii) what the paper is about and what are the main conclusions of the research and (iii), how and based on which specific methodology the authors arrived at their conclusions.

*Table S5. The top 15 words, 2-grams and 3-grams in the trade debate.*

| **top 15 words (trend)** | **top 15 2-grams** | **top 15 3-grams** |
| --- | --- | --- |
| country (-)  model (o)  firm (+)  export (+)  good (o)  market (+)  international (-)  cost (o)  import (+)  data (+)  price (+)  productivity (o)  growth (o)  product (o)  estimate (+) | comparative advantage  per capita  United States  R&D  transport costs  cross country  Heckscher Ohlin  monopolistic competition  equilibrium model  welfare gains  capita income  heterogenous firms  long run  wage inequality  world economy | gains from trade  the United States  terms of trade  factor content of  model of trade  content of trade  in international trade  model of international  per capita income  general equilibrium model  international trade in  of comparative advantage  the exchange rate  the growth of  the terms of |

Notes. Single words are lemmatized. +(-) indicates an upward (downward) linear trend over time, a constant trend (o) is defined as a slope value between -2*10^-4^ and +2*10-4.

*Table S6. The top 15 increasing/decreasing words in the trade debate*

| **top 15 increasing** | **top 15 decreasing** |
| --- | --- |
| firm  export  import  price  quality  data  tariff  markup  estimate  demand  market  sector  gain  cost  liberalization | international  country  world  government  evidence  important  free  multilateral  policy  openness  good  bilateral  convergence  agreement  research |

# Impacts on development studies: the top citing papers

In this section we want to provide detailed information on the top citing papers, i.e. the papers published in WD that most intensively refer to papers in the economic trade debate (Table S7).

*Table S7. The 30 most citing papers in WD. The ranking is based on the sum of the out-degree centralities of the citing papers.*

| **Rank** | **Source** | **Title** | **Trade evaluation** | **Trade impacts** |
| --- | --- | --- | --- | --- |
| 1 | Zhang (2020) | Home-biased gravity: The role of migrant tastes in international trade | Neutral | Econ, Soc |
| 2 | Qian & Yasar (2016) | Export Market Diversification and Firm Productivity: Evidence form a Large Developing Country | Positive | Econ |
| 3 | Francois and Manchin (2013) | Institutions, Infrastructure, and Trade | Ambivalent | Pol |
| 4 | Goel (2017) | Inequality Between and Within Skill Groups: The Curious Case of India | Ambivalent | Econ, Soc |
| 5 | Alvarez et al. (2018) | Does Institutional Quality Matter for Trade? Institutional Conditions in a Sectoral Trade Framework | Neutral | Econ |
| 6 | Yasar (2013) | Political Influence of Exporting and Import-Competing Firms: Evidence from Eastern European and Central Asian Countries | Neutral | Econ, Pol, Soc |
| 7 | De Hoyos & Iacovone (2013) | Economic Performance under NAFTA: A Firm-Level Analysis of the Trade-productivity Linkages | Positive | Econ, Pol |
| 8 | Bresnahan et al. (2016) | Does Freer Trade Really Lead to Productivity Growth? Evidence from Africa | Ambivalent | Econ, Pol |
| 9 | Shepherd (2010) | Geographical Diversification of Developing Country Exports | Positive | Pol |
| 10 | Asuyama (2012) | Skill Distribution and Comparative Advantage: A Comparison of China and India | Positive | Econ |
| 11 | Chandra & Long (2013) | Anti-dumping Duties and their Impact on Exporters: Firm Level Evidence from China | Positive | Econ, Pol |
| 12 | Cheong et al. (2015) | Heterogeneous Effects of Preferential Trade Agreements: How does Partner Similarity Matter? | Ambivalent | Econ, Pol |
| 13 | Beverelli et al. (2015) | Export Diversification Effects of the WTO Trade Facilitation Agreement | Positive | Econ, Pol |
| 14 | Montalabano (2011) | Trade Openness and Developing Countries' Vulnerability: Concepts, Misconceptions, and Directions for Research | Ambivalent | Econ, Pol |
| 15 | Bahar et al. (2019) | Export take-offs and acceleration: Unpacking cross-sector linkages in the evolution of comparative advantage | Positive | Econ |
| 16 | Yasar & Rejesus (2020) | International linkages, technology transfer, and the skilled labor wage share: Evidence from plant-level data in Indonesia | Positive | Econ |
| 17 | Lin & Fu (2016) | Trade, Institution Quality and Income Inequality | Ambivalent | Econ, Soc |
| 18 | Boly et al. (2014) | Diaspora Investments and Firm Export Performance in Selected Sub-Saharan African Countries | Positive | Econ, Soc |
| 19 | Ozler et al. (2009) | History Matters for the Export Decision: Plant-Level Evidence from Turkish Manufacturing Industry | Neutral | Econ |
| 20 | Gallego (2012) | Skill Premium in Chile: Studying Skill Upgrading in the South | Positive | Econ |
| 21 | Alvarez (2007) | Explaining export success: Firm characteristics and spillover effects | Neutral | Econ |
| 22 | Arribas et al. (2009) | Measuring Globalization of International Trade: Theory and Evidence | Positive | Econ, Pol |
| 23 | Felbermayr & Toubal (2012) | Revisiting the Trade-Migration Nexus: Evidence from New OECD Data | Neutral | Pol, Soc |
| 24 | Hoekman & Nicita (2011) | Trade Policy, Trade Costs, and Developing Country Trade | Neutral | Pol |
| 25 | Falciola et al. (2020) | Defining firm competitiveness: A multidimensional framework | Positive | Econ, Pol |
| 26 | Brandi et al. (2020) | Do environmental provisions in trade agreements make exports from developing countries greener? | Positive | Pol, Env |
| 27 | Rougier (2016) | Fire in Cairo: Authoritarian-Redistributive Social Contracts, Structural Change, and the Arab Spring | Positive | Econ, Pol, Soc |
| 28 | Campi & Duenas (2016) | Intellectual Property Rights and International Trade of Agricultural Products | Neutral | Pol |
| 29 | Lee & Ries (2016) | Aid for Trade and Greenfield Investment | Positive | Econ, Pol |
| 30 | Du et al. (2014) | FDI Spillovers and Industrial Policy: The Role of Tariffs and Tax Holidays | Positive | Econ, Pol |

# Discussion and conclusion: analysis of author affiliations

In this section, we finally want to present additional results from our analysis of the institutional affiliation of authors that publish in our sample of DS journals (see Figure S1). More specifically, we compare the distribution of author affiliations across the three (sub-)samples of papers from Figure 1 in the main article. A main advantage of EconLit is that it provides detailed information on author affiliations for every listed article since 1990. For the calculation of (multiple) author affiliations per article we follow the approach of Aistleitner et al. (2022) who assign accurate weightings to each article by first dividing a contribution across authors and, in a second step, relating each author’s share of the contribution to her or his affiliations. In addition, we manually classify each institution into three groups: policy institutions, academic Anglo-Saxon elite universities, and remaining (academic) institutions.

*Figure S1. Analysis of the institutional affiliation of authors that publish in five DS journals.*

References

Aistleitner, M. (2022). Development and Interdisciplinarity: re-examining the “economics silo”. *ICAE Working Paper Series*(139). https://www.jku.at/fileadmin/gruppen/108/ICAE_Working_Papers/wp139.pdf

Aistleitner, M., Kapeller, J., & Kronberger, D. (2022). The authors of economics journals revisited: evidence from a large-scale replication of Hodgson and Rothman (1999). *Journal of Institutional Economics*, 1–16. https://doi.org/10.1017/S174413742200025X

Mitra, S., Palmer, M., & Vuong, V. (2020). Development and interdisciplinarity: A citation analysis. *World Development*, *135*, 105076. https://doi.org/10.1016/j.worlddev.2020.105076
